# Supplementary material for: Autophagy Deregulation in HIV-1-Infected Cells Increases Extracellular Vesicle Release and Contributes to TLR3 Activation
Source: Viruses. 2024 Apr 20;16(4):643. doi: 10.3390/v16040643 (PMC11054313; doi:10.3390/v16040643)
Supplement: Supplementary file 1 [file viruses-16-00643-s001.zip › viruses-2721067-supplementary.pdf]

Supplementary Data

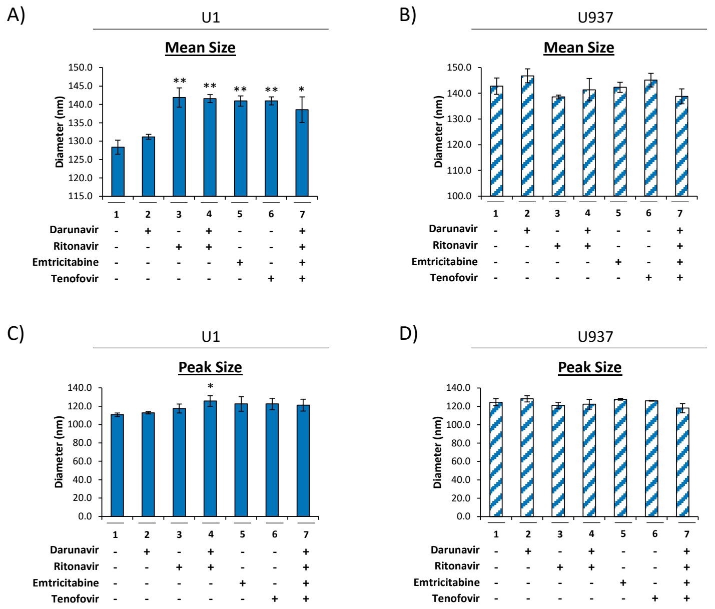

Supplementary Figure 1. Effect of antiretrovirals on EVs from HIV-1-infected monocytes. U1 (HIV-1-infected monocytes) and U937 (uninfected monocytes) cells were treated twice with Darunavir (10 μM), Ritonavir (5 μM), Darunavir/Ritonavir (10 μM/ 5 μM), Emtricitabine (10 μM), Tenofovir (10 μM), or a combined regimen for 5 days in exosome-free media. Supernatants were analyzed using NTA to assess for drug mediated changes in released EV mean diameter in treated U1 (**A**) and U937 (**B**) cells. Additional analysis investigated changes in EV peak diameter from U1 (**C**) and U937 (**D**) cells. Statistical significance was assessed using a two-tailed Student's *t*-test comparing the treated samples (lanes 2–7) to an untreated control (lane 1). $*p < 0.05$, $**p < 0.01$, $***p < 0.001$.

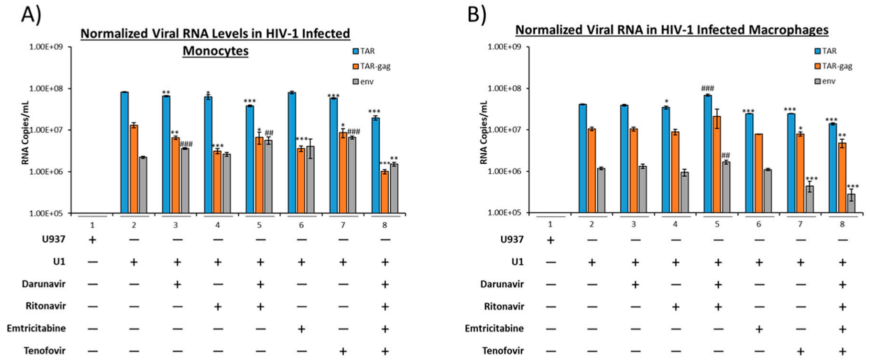

Supplementary Figure 2. Viral RNA decreases with antiretroviral treatment. Levels of viral RNA were assessed from uninfected (U937) and HIV-1-infected (U1) monocytes and macrophages. U1 cells (1×10^6 cells/mL) were treated twice with darunavir (10 μM), ritonavir (5 μM), darunavir/ritonavir (10 μM/ 5 μM), emtricitabine (10 μM), tenofovir (10 μM), or a combined regimen (10 μM pf emtricitabine, tenofovir, darunavir, and 5 μM of ritonavir) for 5 days in exosome-free media. On day 5, the cells were harvested and assessed for viral RNA (TAR, TAR-*gag*, and *env*) normalized to a negative control (A,B). A Student's t-test was used to compare the treated subpopulations to their corresponding un-treated subpopulations. *, $p < 0.05$; ** , $p < 0.01$; ***, $p < 0.001$. Error bars, S.D.

Formatted: Font color: Auto

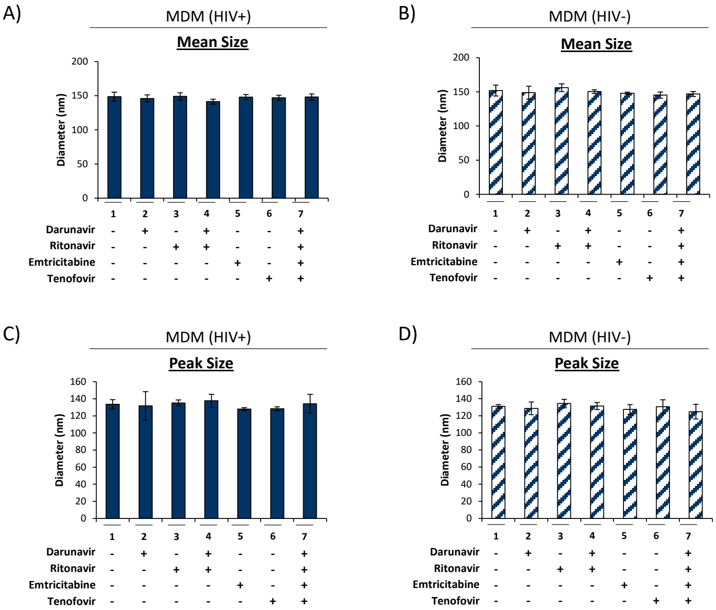

Supplementary Figure 3. Effects of antiretrovirals on EVs from HIV-1-infected monocyte-derived macrophages. U1 (HIV-1-infected monocytes) and U937 (uninfected monocytes) cells were differentiated into monocyte-derived macrophages (MDMs) using PMA (100 nM) over 7 days. Following differentiation, MDMs were treated twice with darunavir (10 μM), ritonavir (5 μM), darunavir/ritonavir (10 μM/ 5 μM), emtricitabine (10 μM), tenofovir (10 μM), or a combined regimen for 5 days in exosome-free media. Supernatants were analyzed using NTA to assess for drug-mediated changes in EV mean diameter in treated HIV+ MDMs (**A**) and HIV− MDMs (**B**). Additional analysis investigated changes in EV peak diameter from HIV+ MDMs (**C**) and HIV− MDMs (**D**). Statistical significance was assessed using a two-tailed Student's *t*-test comparing the treated samples (lanes 2–7) to an untreated control (lane 1). $*p < 0.05$, $**p < 0.01$, $***p < 0.001$.

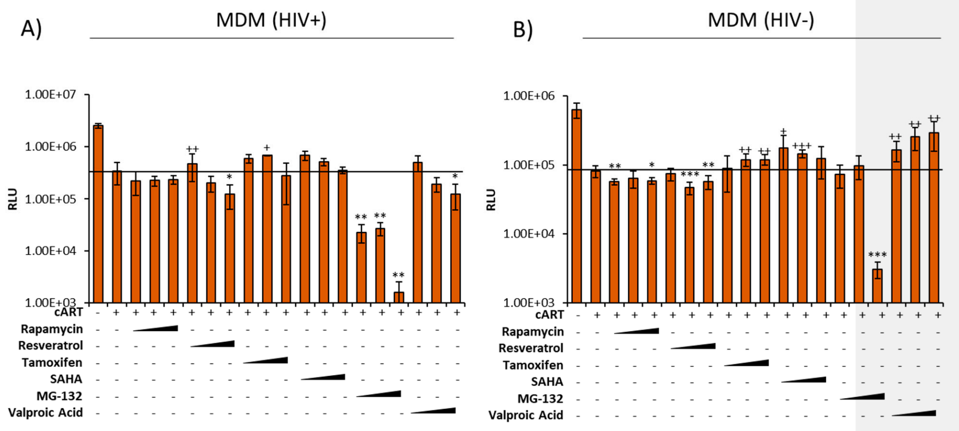

Supplementary Figure 4. Cell viability of MDMs treated with cART and autophagy inducers. Uninfected (U937) and HIV-1-infected (U1) monocytes were differentiated into MDMs using 100 nM PMA over 5 days. MDMs were treated twice with cART (10 μM of emtricitabine, tenofovir, darunavir, and 5 μM of ritonavir) and the autophagy-inducing drugs rapamycin (1 nM, 50 nM, 100 nM), resveratrol (1 μM, 25 uM, 50 uM), tamoxifen (0.1 uM, 2.5 μM, 10 uM), SAHA (0.1 uM, 0.5 uM, 1 μM), MG-132 (0.1 uM, 1 uM, 10 uM), and valproic acid (60 μM, 600 uM, 1 mM) for 5 days. Cell viability was assessed using Cell Titer-Glo reagent. Statistical significance was assessed using a two-tailed Student's *t*-test comparing the samples treated with the autophagy drugs (lanes 2–7) to a control (lane 1). $*p < 0.05$, $**p < 0.01$, $***p < 0.001$.

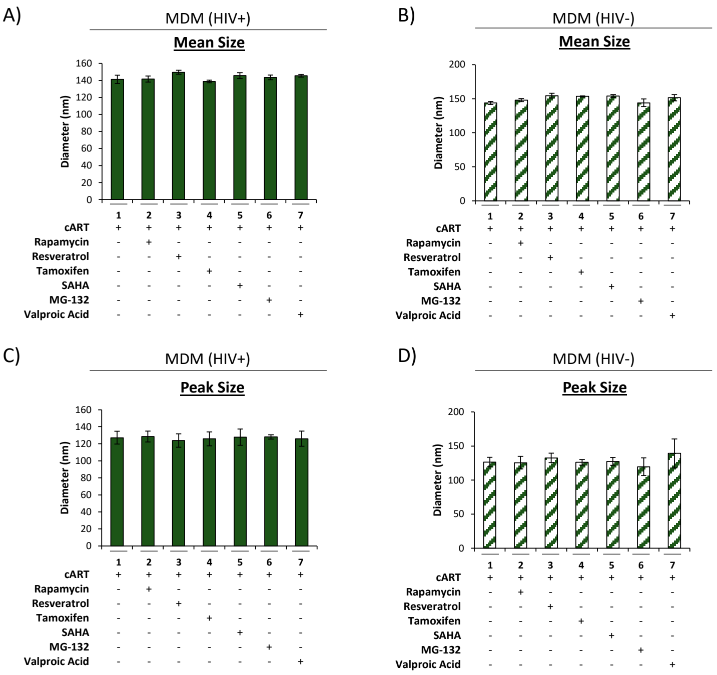

Supplementary Figure 5. Effect of antiretrovirals and adjuvant autophagy compounds on EVs from HIV-1-infected monocyte-derived macrophages. U1 (HIV-1 infected monocytes) and U937 (uninfected monocytes) were differentiated into monocyte-derived macrophages (MDM) using PMA (100 nM) over 4 days. Following differentiation, MDMs were treated twice with cART (10 μM of emtricitabine, tenofovir, darunavir, and 5 μM of ritonavir) as well as autophagy-inducing compounds: rapamycin (100 nM), resveratrol (1 μM), tamoxifen (2.5 μM), SAHA (1 μM), MG-132 (50 nM), and valproic acid (60 μM) for 5 days. Supernatants were analyzed using NTA to assess for drug-mediated changes in EV mean diameter in treated HIV+ MDM (**A**) and HIV− MDM (**B**). Additional analysis investigated change in EV peak diameter from HIV+ MDM (**C**) and HIV− MDM (**D**). Statistical significance was assessed using a Student's *t*-test comparing the samples treated with autophagy inducers (lanes 2–7) to a control (lane 1). $*p < 0.05$, $**p < 0.01$, $***p < 0.001$.

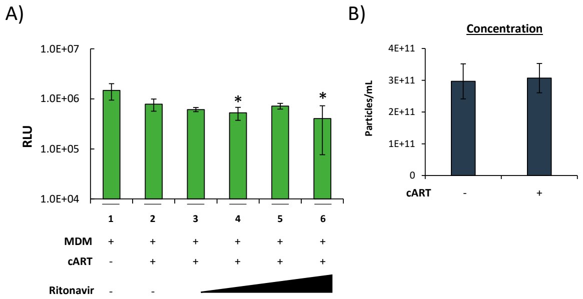

Supplementary Figure 6. Optimization of cART regimen and control of donor cell EV dosing. (**A**) U1 (HIV-1-infected monocytes) cells were differentiated into monocyte-derived macrophages (MDMs) using PMA (100 nM) over 4 days. Following differentiation, MDMs were treated twice with darunavir (10 μM), emtricitabine (10 μM), tenofovir (10 μM), and a titration of ritonavir (0.1 μM, 1 μM, 5 μM, and 10 μM) for 5 days. Viability was assessed post-treatment using a CellTiter-Glo Luminescent Cell Viability Assay. U1 monocytes were differentiated into MDMs using PMA (100 nM) and treated with ± cART (10 μM of emtricitabine, tenofovir, darunavir, and 5 μM of ritonavir) for 5 days. EVs were isolated using ultracentrifugation and equilibrated to control for concentration (**B**). Statistical significance was assessed using a Student's *t*-test comparing the treated samples to an untreated control. $*p < 0.05$, $**p < 0.01$, $***p < 0.001$.

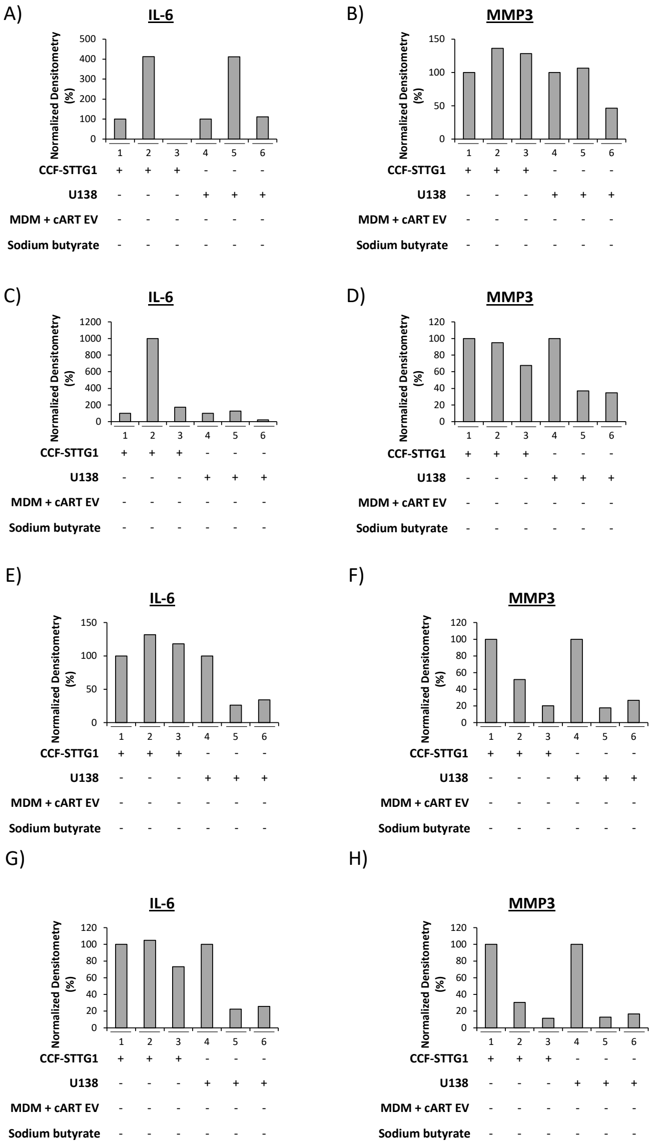

Supplementary Figure 7. Densitometry analysis for EV-induced secretion of inflammation from astrocytes. Densitometry counts of extracellular **A**) 24-hour IL-6 and **B**) MMP3, **C**) 48-hour IL-6, **D**) MMP3, **E**) 72-hour IL-6, **F**) MMP3, **G**) 96-hour IL-6, and **H**) MMP3, as measured using ImageJ software and normalized to actin. The counts are shown as the relative expression of the protein relative to the untreated control (lane 1 set to 100%).

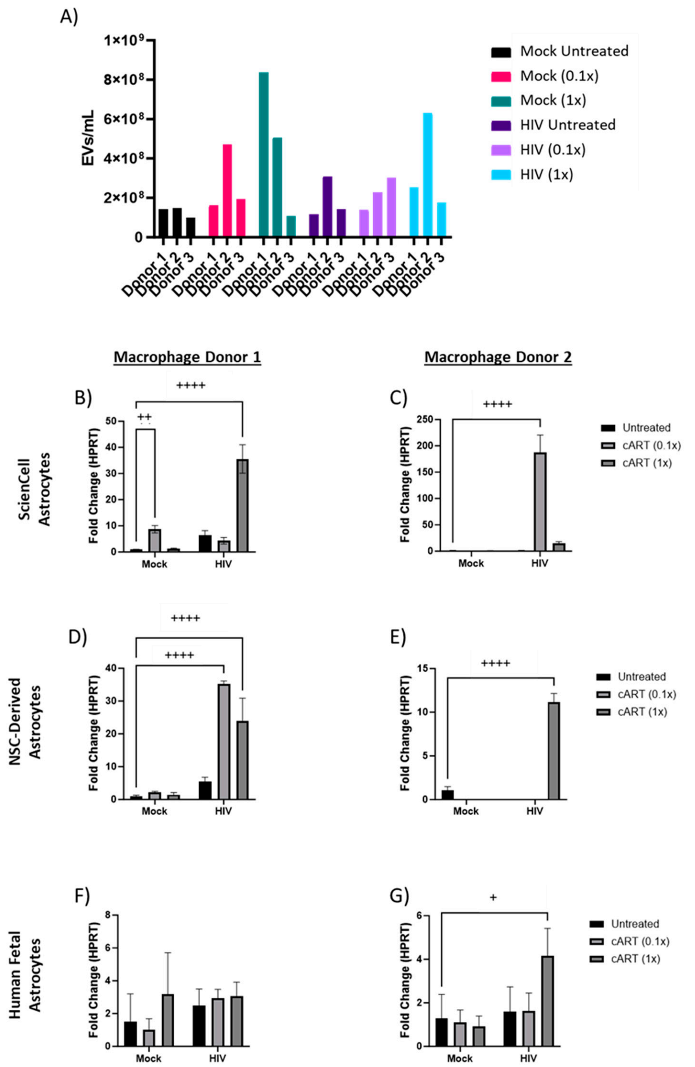

Supplementary Figure 8. The effects of donor EVs on astrocyte inflammation. Primary macrophages were infected with HIV-1 Δ89.6 or mock. The macrophages were then treated twice with cART (0.1× concentration: 0.1 μM of emtricitabine, tenofovir, darunavir, and ritonavir, or 1× concentration: 1 μM of emtricitabine, tenofovir, darunavir, and ritonavir) over 5 days. After incubation, EVs were collected via ultracentrifugation, and EV concentration was measured (A). These EVs were then placed on commercial astrocytes, neural stem cell-derived astrocytes, or human fetal astrocytes for 48 h, after which the cell pellets were collected for RT-qPCR analysis of proinflammatory IL-6 normalized to HPRT (B-G). Statistical significance was assessed using a two-way ANOVA comparing the treated samples to an untreated control. *$p < 0.05$, **$p < 0.01$, ****$p < 0.0001$.

Formatted: Font color: Auto

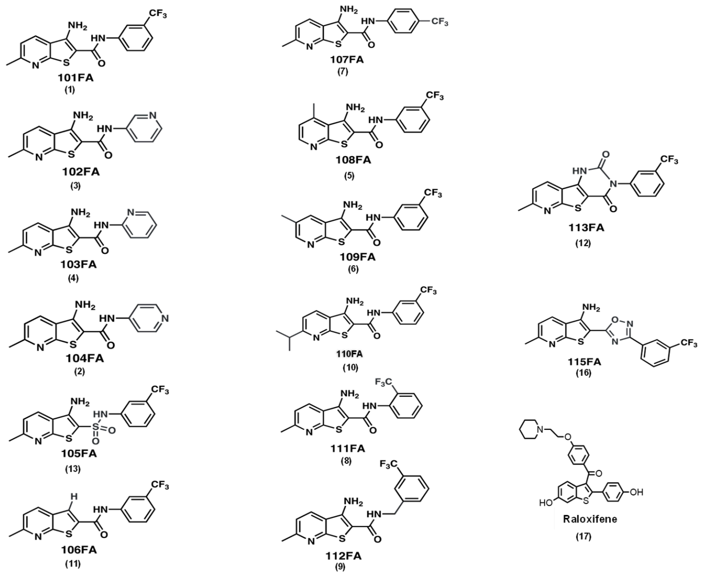

Supplementary Figure 9. HIV-1 TAR-binding ligands used in this study. Numbers in parentheses indicate their designation in the original study of Abulwerdi et al. (54). Raloxifene (#17) was not one of the original TAR binders, but it was also included in this study.

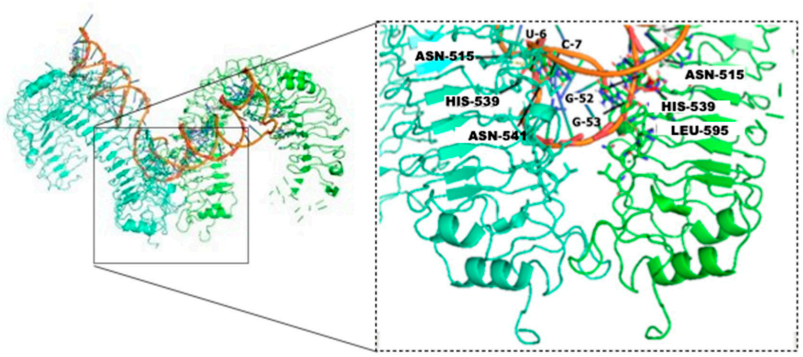

Supplementary Figure 10. Molecular docking of TAR RNA and TLR3. Residues involved in the interaction on the C-terminus of TLR3. His539, an active site C- terminus residue on both TLR3 monomers, interacts with TLR3 at the U6 and G52 nucleotide positions. A known residue, Asn515, also interacts with TAR RNA at the U6 and G52 nucleotide positions on both of the monomers. Other residues, Asn541 and Leu 595, interact with TAR RNA at the C7 and G53 positions, respectively. Docking results were visualized using PyMol (50).
